# Are Antimicrobial Peptides a 21st-Century Solution for Atopic Dermatitis?

**DOI:** 10.3390/ijms241713460

**Published:** 2023-08-30

**Authors:** Manuela Machado, Sara Silva, Eduardo M. Costa

**Affiliations:** CBQF Centro de Biotecnologia e Química Fina Laboratório Associado, Escola Superior de Biotecnologia, Universidade Católica Portuguesa, Rua Diogo Botelho 1327, 4169-005 Porto, Portugal; mmachado@ucp.pt

**Keywords:** AMPs, atopic dermatitis, chronic inflammatory disease, skin infection, barrier disruption, skin microbiome

## Abstract

Atopic dermatitis (AD) is a chronic inflammatory skin disorder that is the result of various environmental, bacterial and genetic stimuli, which culminate in the disruption of the skin’s barrier function. Characterized by highly pruritic skin lesions, xerosis and an array of comorbidities among which skin infections are the most common, this condition results in both a significant loss of quality of life and in the need for life-long treatments (e.g., corticosteroids, monoclonal antibodies and regular antibiotic intake), all of which may have harmful secondary effects. This, in conjunction with AD’s rising prevalence, made the development of alternative treatment strategies the focus of both the scientific community and the pharmaceutical industry. Given their potential to both manage the skin microbiome, fight infections and even modulate the local immune response, the use of antimicrobial peptides (AMPs) from more diverse origins has become one of the most promising alternative solutions for AD management, with some being already used with some success towards this end. However, their production and use also exhibit some limitations. The current work seeks to compile the available information and provide a better understanding of the state of the art in the understanding of AMPs’ true potential in addressing AD.

## 1. Introduction

To understand the dos and don’ts of peptide applications in human skin one must first understand not only the skin’s role and characteristics, but also what makes skin diseases, such as atopic dermatitis, so hard to treat and manage.

Considered by most to be the largest organ in the human body (as it possesses a surface area of approximately 2 m^2^) the skin’s main function is to be a physical barrier, which protects us from the external surrounding environment [1]. While this barrier function is mostly physical, there is also a “gatekeeper” aspect to it, as the combination of the cells and matrix elements that constitute the skin acts as a “custom agent” that permits or denies microorganism colonization of the skin surface and determines which compounds can migrate through the various layers and either reach the bloodstream or leave the body [2].

**Figure 1:**
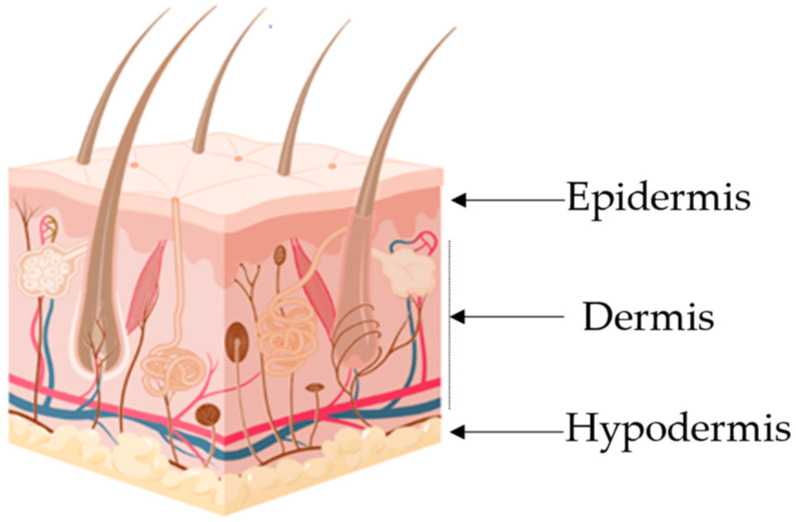
Schematic 3D representation of the human skin. Image produced using Biorender.

From a structural perspective, the skin is constituted of three major layers, which are, from inside out, the hypodermis, the dermis and the epidermis (Figure 1). As can be seen from Table 1, each layer comprises different cellular constituents having different functions and is home to different structures, such as blood vessels in the hypodermis and dermis, mechanoreceptors in the dermis, and a stratified keratinized epithelium in the epidermis [1,2].

When one considers the skin complexity a compound’s biological activity will be mainly influenced by two layers, the epidermis followed by the dermis. The reason for this is that, within these layers, all physical and chemical resistance to compound penetration, immunological response to outside factors, and the management of microbial colonization and infection resistance is provided [3,4]. When alterations to these layers occur, there is a window for the appearance of abnormal conditions within the skin and the development of hard-to-treat-and-manage diseases, such as AD.

## 2. Atopic Dermatitis

Atopic dermatitis is one of the most common and recurrent chronic non-infectious skin inflammatory diseases, which is characterized by a persistent itching sensation in the skin. It is a skin disorder that usually appears in early childhood (about 80% of the cases) and is reported to affect 15–20% of children. Although ca. 70% of paediatric patients outgrow the disease, the prevalence in adults remains around 1–3%, although the figures vary greatly from country to country [6,7,8,9]. AD’s worldwide prevalence is rising, with two- to three-fold increases in incidence in industrialized countries being reported for the last decades, having reached a plateau only in countries where its prevalence is highest [7,10]. In contrast to most allergic diseases, AD has a high social and economic impact. The chronic skin inflammation with continuous itching leads to skin thickening, lichenification and overall discomfort. This will lead to a compromise in sleep patterns, which have social consequences and create economic burdens. All of these social and economic impacts are what makes AD a disease with a high toll on patients and their families [11,12].

From a clinical standpoint, AD belongs to the spectrum of atopic disorders, such as allergic asthma or food allergies. Coincidently, these are companion diseases of AD patients due to the commonly denominated “atopic march”, a curious denomination given to the range of allergic disorders that, in later years, manifest in AD patients [13,14,15]. Atopic dermatitis clinical diagnostics are characterized by eczema-like eruptions, papules, exudative lesions, and various degrees of skin dryness. In addition, there are several comorbidities, such as skin infections and cardiovascular and neuropsychiatric disorders, which have also recently been associated with AD, despite the mechanism behind these associations being still unknown [11,16,17,18,19,20]. As with most atopic diseases, there is a strong environmental link with immunoglobulin E (IgE)-related environmental responses, with this factor being one of the primary drivers of AD outbreaks [21]. However, AD is a complex multifactorial disease that cannot be attributed to one single cause.

From a pathophysiologic standpoint, AD results from several genetic defects that potentiate the immune response and disrupt the skin barrier. The most reported mutations are associated with filaggrin (FLG) production, which have been recognized as some of the most relevant, with various authors reporting that up to 60% of Europeans with AD exhibit alterations in FLG expression [11]. Another defect which plays a key role in AD are tight junctions (TJs) mutations and alterations. These are structures that have a connection role between cells, exist in every human epithelium, and have various roles depending on the tissue they are located in (e.g., homeostasis control in the central nervous system or impeding the penetration of pathogens in the intestine) [22,23]. In healthy skin, TJs are part of the mechanism for managing cellular differentiation, proliferation and cascading processes involved in maintaining and managing skin homeostasis and permeability [24,25]. On the other hand, in AD patients, TJs are normally dysfunctional and contribute to the irregular barrier function of the skin. They appear to impede stratum corneum (SC, the outermost layer of the epidermis) formation due to increased pH values and, among other effects, affect the processing of polar lipids and profillagrin, both of which are critical for SC formation. All these alterations lead to increased permeability to exogenous material and bacteria, which results in an increased inflammatory condition and a vicious circle, where the barrier dysfunction potentiates the skin’s inflammatory response. Interestingly, the TJ dysfunction is not directly affected by the FLG mutation, with both mechanisms appearing to be independent [26,27].

In addition to these two main alterations, several other structural proteins have been described as downregulated in AD, such as desmogleins, desmocolins, involucrin and keratins [28,29,30,31,32]. These alterations to expression and mutations again result in compromise of the skin’s shield function, increased transepidermal water loss (i.e., higher dehydration), and an increased exposure to external toxins and allergens that translates into activation of the local immune system.

Of all the identified changes, the FLG mutation is a particularly interesting one when seeking to understand the cascade of potential problems that arise from a single mutation: (1) it disrupts the production of moisturizing factors in the skin’s stratum granulosum; (2) it disrupts lamellar body secretion and, therefore, alters the composition of the SC; (3) it arrests the metabolic pathway part-way through resulting in the absence of acidic metabolites. This translates into an upswing of the SC pH and allows for an increase in proteolytic enzyme activity (increased desquamation) and the proliferation of *Staphylococcus aureus* (*S. aureus*), a bacterial species whose predominance in skin flora has been strongly associated with AD flares [17,33,34,35].

While the pathophysiology of AD is mainly attributed to the above-referenced FLG and TJs disruptions it is in fact a very complex process. So complex that, when talking about this topic, one must consider four fields or target areas. The first is the environmental area, the second is the skin microbiome, the third is the epidermal barrier, and the fourth is the immune/inflammatory response [36,37,38]. The first consists of the sum of all the environmental (the “exposome”) influences that may affect AD pathogenesis and progression. It includes factors such as air pollutants, allergens and microorganisms (bacteria, viruses and fungi) and spans all domains of everyday life, including an individuals’ diet and behaviour [39]. The skin microbiome (second field/target area) relates strongly to *S. aureus*-exacerbated skin colonization and infection, which, in conjunction with imbalances in skin microbiota in AD lesions, strongly influence AD progression in patients, particularly due to the expression by *S. aureus* of several superantigens, cytolytic α- and δ-toxins, and clumping factor B, which allows it to adhere to deformed corneocytes in AD skin, and proteases which further degrade the epidermis [21,40,41]. Epidermal barrier (third field/target area) disruption is a hallmark of AD and is mostly due to a combination of two factors: first, mutations or alterations in the genes encoding the structural-filament-associated protein filaggrin that binds to keratin fibres in epithelial cells [42], and, second, the overexpression of inflammatory cytokines, such as IL-13, which will further disrupt the epidermal layer and impair or dysregulate epidermal barrier function, leading to increased skin permeability [43,44]. Last, but not least, we have the activity of the immune/inflammatory response as, in AD patients, there is an overall dysregulation or impairment of genes involved in the innate response, which leads to a lack of production of the cytokines involved in fighting pathogen skin colonization [44,45]. Similarly, dysregulation of the adaptative immunity in AD patients, particularly in the response of CD4^+^ T-helper cells, leads to the overexpression of IL-17 and IL-22 cytokines, both of which have been associated with acute and chronic AD lesions [45,46].

Considering the pathophysiology of AD, it would be natural to expect that its treatment would involve a multifaceted approach capable of mitigating or ameliorating several facets of the disease, with a multitude of approaches and options for patients. However, this could not be further from reality. As AD is considered as being only a skin disorder, the treatments follow the paradigm “one-size-fits-all”, with solutions being very limited, and an almost recipe-like approach being followed by physicians all over the world [21]. In fact, most AD patients’ treatment has, as a first line of approach, anti-inflammatory treatment, more particularly, topical corticosteroids or topical calcineurin inhibitors, in conjunction with an antimicrobial ointment to help with *S. aureus* control [47,48]. For more severe cases, this is normally followed by the use of ultraviolet light and potent cocktails of immunosuppressant drugs, such as ciclosporin A, methotrexate and mycophenolate mofetil [21,47,49]. These regimens, while mostly effective, are not without costs, as several side-effects related to cutaneous application of immunosuppressants are well-described in the literature [50,51]. Thus, there has been an increasing demand for alternative solutions to AD treatment.

Currently, there are already some therapeutic alternatives being tested, such as Janus kinase inhibitors (e.g., Delgocitinib and Ruxolitinib), phosphodiesterase-4 inhibitors (e.g., Crisaborole and Difamilast) and aryl hydrocarbon receptor agonists (e.g., Tapinarof), all of which, through topical application, target the inhibition of enzymes, receptors or transcriptional factors involved in AD [52,53,54]. Another option is the use of human monoclonal antibodies (mAbs), which are also already being applied in AD patients via systemic application [55,56]. However, both of these alternatives are used mainly for moderate to severe cases and, thus, are not suitable for everyday applications [57]. Thus, alternatives are still required for the daily management of AD. Among the natural alternatives currently being studied, antimicrobial peptides (AMPs) are some of the most promising.

## 3. Antimicrobial Peptides

By definition, AMPs are small molecules that are widely present in nature and are part of the immune response of most human inflammatory responses. They are generally constituted by 100 amino acid residues or less and have a positive net charge and amphiphilic structure, which provides these molecules enhanced biological potential due to their natural strong interaction with hydrophobic surfaces and membranes [58]. In terms of their characterization, AMPs can be divided according to their structure (α-helical, β-sheets, extended peptides and loop peptides) and covalent bonds (class I to IV) [59]. Their typical characteristics are described in Table 2.

In the context of the epidermis, naturally produced AMPs play a crucial role in the maintenance of a healthy skin microbiome through modulation of the microbiota composition, proliferation and death, and, thus, also play a role in the metabolites produced by them. Produced within the epidermis, primarily in keratinocytes, this family of ca. 20 peptides not only exhibits antibacterial activity, but these peptides also exert immunomodulatory effects as they activate various cell-related functions, such as migration and proliferation, regulate cytokine production and help maintain the skin’s barrier function, and play critical roles in both innate and adaptative immunity [75]. In this family, the most studied AMPs are defensins, LL-37, RNase 7, psoriasin and dermcidin, with most of these AMPs being expressed at basal or low levels in healthy skin, with the only exception being the β-defensin AMPs which are crucial for the homoeostasis of healthy skin and its protection against infections [76]. For the ones that are expressed at basal levels, their production is normally induced in injury, inflammation or infection scenarios (Table 3) [12,75,76].

Disruptions to AMP production or activity have been shown to contribute significantly to the increased susceptibility of AD patients to skin infections by fungi, bacteria and viruses and, thus, are perceived as an important factor in AD pathophysiology [11]. In fact, in AD patients AMPs such as Dermcidin, LL-37 and β-defensins are un-der-expressed, a fact that has been shown to directly influence the progression of AD. This is due to these AMPs being involved in numerous adaptative and innate immune responses (through the recruitment of a broad range of leukocytes) among which are: (i) the regulation of the itch sensation; (ii) secretion of IL-31 from mast cells induced by β-defensins and LL-37; (iii) under expression of AMPs, due to overexpression of Th2 cytokines, hampering S. aureus killing in keratinocytes [12,16]. On the other hand, under AD conditions, some AMPs are overexpressed, such as Rnase7, leading to imbalances in the skin microbiota. There is also the curious case of β-defensins and LL-37, which can be the cause of their own inhibition, as they recruit and activate a broad range of leukocytes. This leads to the production of IL-4, IL-13 and IL-31, the major interleukins involved in the development of AD, thus creating a perfect inflammatory environment for the pathogenesis of AD and their own inhibition [83].

**Figure 2:**
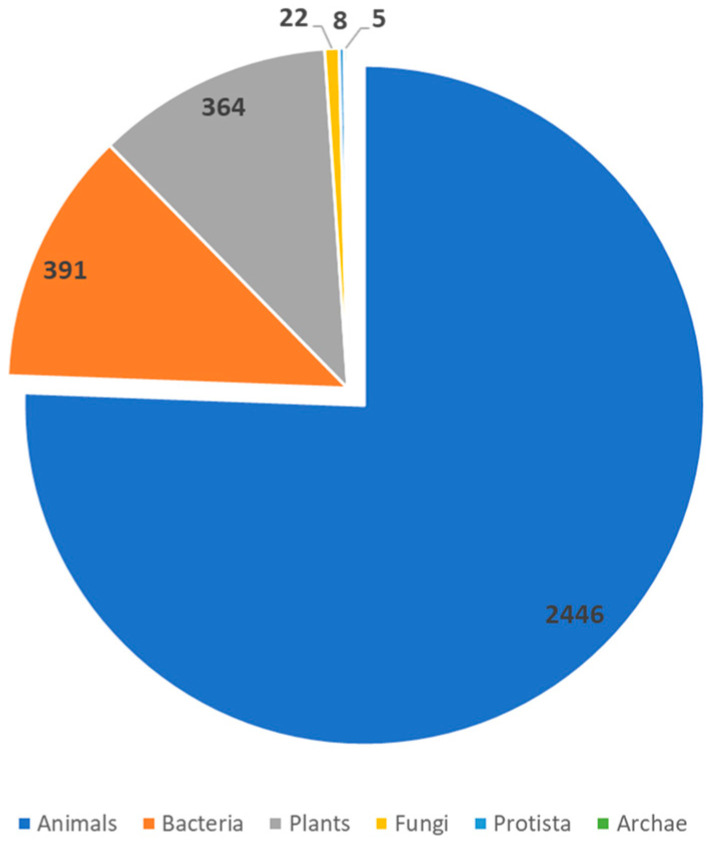
Distribution of known AMPs across the different sources. Data accessed from the Antimicrobial Peptide Database https://aps.unmc.edu (accessed on 15 August 2023).

With this clear influence upon various and critical factors involved in AD pathogenesis, AMPs have risen to prominence as valid alternatives for the treatment/management of AD. In recent years, research has centered on the use of AMPs either as an alternative to antimicrobials or as a pharmaceutical agent capable of exerting immunomodulatory activity, with several natural and synthetic sources being explored. A total of 3324 AMPs has now been registered with the Antimicrobial Peptide Database from sources of the six traditional kingdoms (bacteria, archaea, protists, fungi, plants and animals) (Figure 2) [84].

From a mechanistic standpoint, the application of AMPs as pharmacodynamic agents is dependent on a variety of factors (such as peptide concentration, tissue location, the local environment and the target pathogen) that one must consider when evaluating AMP potential application in skin and AD in particular [85]. While there are some disadvantages regarding the clinical application of AMPs, like their rapid degradation by esterases or aminopeptidases and their low stability in vivo, there are also advantages to their use. These include their capability for penetrating deeper skin layers, as shown by their presence in viable skin even 24 h after application, and the fact that AMP-based treatment provides opportunities for interventions that closely resemble natural pathways and represent more of a replacement therapy than conventional treatment. In this regard, they supplement patients with AMPs in locations where their endogenous levels are low or absent and, consequently, are less likely to promote adverse responses [84,86]. Furthermore, even the previously mentioned disadvantages do not represent a limitation to AMP application in AD management, as, nowadays, there are several strategies to enhance their bioavailability and overcome their limitations. These include, penetration enhancers, encapsulation, or even chemical modification to increase AMP permeability through the skin [87].

### 3.1. Human AMPs

One example of a human source AMP is AMP-IBP5. This is derived from the insulin-like growth factor binding protein 5 (IBP-5), which is obtained through proteolytic cleavage and has been found to be expressed in various cells, including keratinocytes and fibroblasts. This AMP has been reported to accelerate diabetic wound healing through protection against glucotoxicity and increased angiogenesis. Furthermore, it was reported as being capable of inducing the proliferation of keratinocytes and fibroblasts through the activation of the low-density lipoprotein receptor-related protein 1 (LRP1), a receptor which is mainly expressed in the stratum granulosum in the epidermis and in dermal fibroblasts [88,89,90]. This AMP has been shown by Nguyen, Peng, Trujillo-Paez, Yue, Ikutama, Takahashi, Umehara, Okumura, Ogawa, Ikeda and Niyonsaba [16] to be capable of improving the skin’s barrier function, as it upregulated TJ-related protein (claudin-1, -4 and -7, occluding and ZO-1) expression, with an even distribution of the expressed proteins throughout the epidermis, and led to increases in barrier function over a 48 h period after its application. Additionally, a cellular model showed that this AMP was capable of ameliorating IL-4/IL-13-driven TJ barrier dysfunction. From a mechanistic standpoint, the reported data showed that this peptide activated the atypical protein kinase C ζ and ras-related C3 botulinum toxin substrate 1 expression. This has been shown to be linked to TJ barrier function and to the activated LRP1 receptor.

Another example of a human-derived or obtained AMP is DPK-060, or, as it is also known, GKH17-WWW. This is a chemically synthesized AMP, structurally derived from kininogen, a protein found in humans, and to which three tryptophan residues were added to the C-terminal side. This grants this AMP increased resistance to enzymatic degradation without added cytotoxic effects. From a functional standpoint, this AMP has strong antimicrobial activity against various Gram-positive and Gram-negative bacteria, including methicillin-resistant *S. aureus*. A clinical trial where a DPK-060-formulated ointment was applied to AD patients showed that this compound was well-tolerated and led to a reduction of 94 to 99% of microbial load in lesions after 14 d of application [59,91].

### 3.2. Microbial AMPs

Bacterial AMPs are short peptide bacteriocins that inhibit microorganism growth. More recently, peptide molecules from this class that can inhibit bacterial quorum sensing have also gained attention with a potential focus being on their application in AD treatment [12,92].

Short peptide bacteriocins (SPBs) are ribosomally synthesized peptide antimicrobials with a molecular weight of less than 5 kDa, which possess a narrow but potent spectrum of antibacterial activity, with little to no known bacterial resistances. They are capable of inhibiting bacterial growth through different mechanisms, such as interfering with cell wall biosynthesis, membrane disruption or inhibiting protein synthesis in the 50s ribosomal subunit [93]. Traditionally the antimicrobial activity of SPBs is targeted towards closely related bacteria and, thus, a directed response, where one microorganism is inhibited and another is not, can be achieved. One example of this is Subtilosin A, an SPB produced by *Bacillus subtilis*, which is capable of exerting strong inhibitory activity against *Streptococcus pyogenes* at a very low concentration (1.25 µg/mL), but against *Streptococcus gordonii* had an MIC value of 83.25 µg/mL [94]. This discrepancy may be key to targeted activity against skin pathogens in the skin microbiome and *S. aureus,* in particular. Examples of this potential can already be found in the literature. One is the NAI003 peptide, which has already passed phase 1 clinical trials and showed selective activity against *Cutibacterium acnes* (*C. acnes*) and not against commensal skin microorganisms. Curiously, the same *C. acnes* is responsible for the production of the SPB cutimycin, a peptide which is capable of inhibiting *Staphylococcus* species growth. Another is the SPB *Lactocillin*, a peptide isolated from the vaginal commensal microorganism *Lactobacillus johnsonii* PF01, which has showed the capacity to inhibit pathogens from colonizing the skin [95,96]. One example, which clearly illustrates the curious nature of AMPs produced by skin microorganisms, is the targeted activity of various staphylococci species against *S. aureus*, as *Staphylococcus epidermidis* produces epidermin or the hominicin produced by *Staphylococcus hominis*, with both AMPs being extremely effective against *S. aureus* [12,96,97,98].

The other already touched field of application is quorum sensing inhibition, or, more particularly, targeting the quenching of *S. aureus* quorum sensing signal molecules. Quorum sensing is, by definition, a cell-density-dependent phenomenon that enables bacterial cell-to-cell communication through extracellular chemical signals, which are identified by cell receptors, leading to the activation of genes for the production of certain metabolites within a cellular community and, thus, regulating their growth and behaviour [99]. In the case of *S. aureus* and AD the capacity of SPBs to inhibit this quorum sensing mechanism may be of particular importance, as the agr quorum sensing system of *S. aureus* as the activator of the kinase receptor AgrC initiates a cascade that leads to the transcription of several virulence factors associated with AD pathogenesis [100]. This has been demonstrated in the work of William and his co-workers who isolated and identified an SPB from *S. hominis* which was capable of inhibiting *S. aureus* quorum sensing and limited *S. aureus*-mediated epidermal proteolysis and inflammatory response [37]. Another example is the work of Brown et al. [101] where it was demonstrated that an SPB obtained from *Staphylococcus simulans* was capable of reducing MRSA-related dermonecrotic and epicutaneous skin lesions in mouse models.

A further example of bacterial AMPs’ possible application in AD treatment was described by Van Hemert et al. [102] who showed that AMPs produced by *Lactobacillus plantarum* were capable of modulating cytokine production in PBMCs, with an increase in the production of the anti-inflammatory cytokines IL-10 and IL-12 by over 10-fold.

### 3.3. Animal-Sourced AMPs

One example of animal-sourced AMPs with AD application is the 12-amino-acid-long peptide Omiganan, which is derived from indolicidin, an AMP obtained from the cytoplasmatic granules of bovine neutrophils, which has been used as a local treatment for AD due to its antimicrobial and antibiofilm capacity against skin bacteria and fungi [59,103]. The results obtained from clinical trials with this peptide showed that this peptide had an effective in vivo antibacterial effect in patients with mild to moderate AD, and that, with treatment, their microbiome shifted from lesional to non-lesional, with a decrease in the presence of staphylococci and an increase in bacterial diversity [104].

Another example of an animal-based AMP can be found in the work of Fei et al. [105] which showed that the AMP Brevinin-1E-OG9c-De-NH2, derived from the frog AMP brevinine, had triple potential as a potential AD agent, as it had strong in vitro and ex vivo antimicrobial activity against *S. aureus*, was capable of inhibiting *S. aureus* biofilm formation, and attenuated the inflammatory response in keratinocytes induced by lipoteichoic acid and lipopolysaccharide.

## 4. AMPs in Clinical Trials

As with all pharmaceutical-related applications, clinical trials are the last and crucial step that a formulation must pass to be European Medicines Agency (EMA) or Food and Drug Agency (FDA) approved for human application [106]. The purpose of these trials is twofold—first, to validate in vivo the biological activity verified in vitro; second, to establish a safety profile of the formulated peptides so correct dosages and side-effects can be correctly ascertained [107].

As can be seen in Table 4, there are numerous examples of AMPs under consideration with a vast array of applications being considered, with their status ranging from phase I to III. The success rate of these trials and the posterior application of AMPs as therapeutic agents is variable as, while there are currently several AMPs approved for clinical use, such as ghrelin, nisin, gramicidin or daptomycin, there are others, such as CZEN-002 or NVB-302, which have been discontinued as they failed in their clinical trials [106,107,108,109]. The causes for these failures are usually unknown and unexpected as all AMPs are validated by various in vitro, in silico and in vivo models before reaching this stage [108]. One example of such failures is the Friulimicin AMP, which was terminated due to unfavorable kinetics in healthy volunteers and another is the Murepavadin AMP as, in a stage III trial of patients with nosocomial pneumonia, it caused higher than expected kidney injuries [108].

Of the examples presented in Table 4, it is interesting to see that only Omiganan targets AD-related factors directly. In fact, a cursory analysis of the table shows that most AMPs target bacterial growth, a clear reflection of their namesake, and which has directed research efforts over the past years. When one considers this, the scope for AD-related applications grows immensely as various AMPs in clinical trials target AD-critical aspects, such as bacterial skin infection and, more particularly, *Staphylococcal* growth.

## 5. Limitations to AMPs Usage

When considering the application of AMPs in AD treatment, there are clear limitations that have been previously identified, such as their rapid degradation and their low stability in vivo [86], but there are also other factors that must be considered.

One of the major limitations associated with AMP applications is that, despite their generally positive safety profile, these peptides are highly capable of interacting with eukaryotic cell membranes. These interactions may lead to the disassembling of the membranes and consequent cytotoxicity. When this happens in erythrocytes, hemolysis occurs [146].

When one considers the application of AMPs as antimicrobial agents for the targeted control of *S. aureus*, two limitations have been identified: first, not all patients have *S. aureus* imbalance/colonization in their microbiota, and, second, imbalance in the direction of other staphylococci, such as *S. epidermidis*, has been shown to be as deleterious towards AD patients as these microorganisms produce proteases that damage the host [147]. Furthermore, most microorganisms are capable of developing defense mechanisms against AMPs, thus creating two additional limitations to AMP usage: the development of bacterial resistance to the compounds and variable efficacy of the AMPs [107].

For human-derived AMPs, various limitations may hamper their future applications, but the main one is that they have to be produced using biotechnology-based resources that can be scalable to industrial settings. This by itself is a major hurdle due to the associated high costs. In fact, Koo and Seo [107] stated that AMP production cost varies between USD 50 and 400 per gram of peptide, a value vastly superior to the USD 0.80 per gram reported for the production of the aminoglycoside antibiotic [108]. As for the current biological alternatives, such as mAbs, it is not possible to perform a like-for-like comparison as there is no information regarding their cost per gram. However, as Eichenfield et al. [148] showed, current mAbs treatments in the U.S. have an average annual cost of USD 36.505 and may reach values as high as USD 150.000 [149]. Considering these values, it is possible that future AMP-based solutions may offer competitive pricing against current biological treatments. To do so, the cost limitation of AMPs must be overcome. To that end, alternatives based in biotechnology-based approaches, such as solid-phase peptide synthesis or fermentation systems, in conjunction with the application of machine learning to speed along the translation of the information from a laboratory to an industrial setting, are being applied [150,151]. However, the drawback of these methods is the lack of standardized approaches that leads to a lack of reliable procedures to produce AMPs in the high yields required to make them economically feasible [152].

In addition to all the previously identified limitations, the last major hurdle which AMPs have to overcome to be applied is a regulatory one. In fact, of the over 3324 AMPs found and studied, many are not suitable for pharmacological application in their natural state and very few are EMA- and FDA-approved for AD-related applications as most AMPs lack fundamental information, such as pharmacokinetic information and significant information regarding their potential secondary effects [63,153].

## 6. Conclusions

When one looks into AMPs and their role in AD, it is only natural that they have risen as natural alternatives to the traditional and sometimes ineffective treatment of AD. They are already present in the system and their imbalance is part of the pathology, so, in a logical step, their replenishment should help fight AD and help re-establish homeostasis in the skin of AD patients. While this concept by itself has merit, as shown by the clinical assays that showed that these peptides have a real capacity to influence the outcome of AD patients, there are clear real-world limitations to their full-scale application at this time. Of the existing limitations, the major one is a source problem as, while human-derived AMPs are the clear-cut first choice, there are problems associated with their synthesis, production upscaling and associated cost. When considering alternative secondary sources, such as animal or bacterial sources, there are clear limitations associated with their costs and production, but there are differences between these sources. For animal-based AMPs, there are secondary or deleterious interactions that must be accounted for due to their non-human nature, a limitation that is not normally present for bacterial AMPs.

Last, but not least, there are the various limitations associated with AMP usage already which can be circumvented and bypassed, either through chemical modification of the application of delivery vehicles for the AMPs or through compiling extensive safety and activity dossiers to garner EMA and FDA approval.

So, in conclusion, while AMPs have already shown themselves to be natural and viable alternatives to conventional AD treatment regimens, and pharmacological solutions are already being explored and validated, there is still a way to go to enable AMPs to become cost-effective mainstream solutions for AD treatment.

## Figures and Tables

**Table 1:** Major constituents and functions of the different skin layers.

Layer	Major cellular Constituents	Major Functions	References
Hypodermis	Adipocytes, fibroblasts, endothelial and muscle cells	Insulation, mechanical integrity, support, conductance of vascular and neural signals	[1,2]
Dermis	Endothelial cells, fibroblasts, Langerhans and muscle cells	Mechanical integrity, support, thermal barrier, energy storage, protection from physical injury	[2,3]
Epidermis	Keratinocytes, melanocytes, Langerhans and Markel cells	Outermost barrier, immune function, protection from oxidative and mechanical stress	[4,5]

**Table 2:** Classification of AMPs according to their structure and bonds.

	Structure		
Class	Characteristics	Examples	References
α-helical	Contains <40 amino acids and forms an α-helical secondary structure in a non-polar environment. Antimicrobial activity correlates with α-helical content; these AMPs are mostly membrane disruptive and exhibit activity against fungi, viruses, bacteria, and even drug-resistant pathogens.	Cathelicidins and Magainins.	[60,61,62]
β-sheets	Contain conserved cysteine residues which form disulphide bonds between anti-parallel strands with helical fragments. Target Gram-positive and negative bacteria and complex with bacterial lipopolysaccharides.	Defensins and Tachyplesins.	[63,64,65]
extended peptides	The presence of glycine, histidine, arginine, and tryptophan instead of a structural pattern is the defining characteristic. Engage with membrane lipids and produce hydrogen bonds and van der Waals interactions.	Histatins and Indolicidin.	[61,66]
loop peptides	Distinguished by a single bond (disulphide or amide), which leads to a loop structure. Shows activity against a broad range of Gram-positive and Gram-negative bacteria. Only 1 AMP has been identified with this structure and is constituted by 21 amino acids.	Thanatin	[67,68]
	**Covalent Bond**		
**Class**	**Characteristics**	**Examples**	**References**
I	Linear or open chains with only one chain. No bonding registered.	Magainin and Cecropins.	[69]
II	Side-chain–side-chain with either one or two chains. Single chains with no bonding or two chains with disulphide bonds.	Enterocin L50 and Geobacillin I.	[70,71]
III	Side-chain–backbone bonding pattern with only a single chain present. Bonding is through either amide, ester or thioether bonds.	Capistruin and Huazacin.	[72,73]
IV	A backbone-to-backbone bonding pattern with only one chain present. Bonding through amide bonds.	RTD-1 and Kalata B1.	[74]

**Table 3:** Examples and functions of AMPs produced in human skin. Crystalline structures obtained via accession of the Protein databank.

AMP	Expression Conditions	Major Functions	Crystaline Structure	References
Defensins	β-defensins expressed continuously; other defensins expressed by infection, injury, or pro-inflammatory cytokines	Antimicrobial defense of skin, synergistic effect with LL-37, potent *Candida albicans* and anaerobic pathogen inhibitor	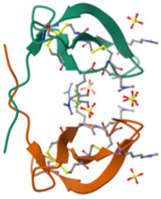	[75,76]
LL-37	Induced by injury and inflammation	Broad antimicrobial activity, chemotactic capacity to recruit neutrophils, T- and mast cells and monocytes. Promotes angiogenesis	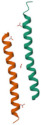	[12,16]
Psoriasin	Induced by inflammatory conditions and barrier disruption	Immunomodulatory properties and strong inhibitor of *Escherichia coli* and other bacteria	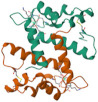	[77,78]
Rnase7	Induced by pro-inflammatory cytokines	Strong inhibitor of *S. aureus*	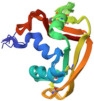	[79,80]
Dermcidin	Expressed in sweat glands; not found in keratinocytes	Active against *S. aureus* and *C. albicans*	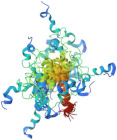	[81,82]

**Table 4:** Examples of AMPs which underwent and are undergoing clinical trials.

AMP Name	Clinical Trial ID	Phase	Target	Reference
AP-214	NCT00903604	II ^a^	Post-surgical organ failure	[110]
C16G2	NCT03004365	II ^c^	*Streptococcus mutans*	[111]
CZEN-002	NCT03145220	II ^a^	Antifungal	[112]
Daptomycin	NCT01922011; NCT00093067; NCT01104662; NCT02972983	III/IV ^c^	Skin infection/bacteremia	[113]
Delmitide (RDP58)	ISRCTN84220089	II ^c^	Inflammatory bowel disease	[114]
DPK-060	NCT01447017; NCT01522391	II ^c^	Acute external otitis, topical treatment of microbial infections	[91]
EA-230	NCT03145220	II ^d^	Sepsis/renal failure	[112]
Friulimicin	NCT00492271	I ^a^	MRSA/pneumonia	[115]
Ghrelin	NCT00763477	II ^c^	Chronic respiratory infection	[116,117]
Gramicidin	NCT00534391	III ^d^	Infected wounds and ulcers	[118]
GSK1322322	NCT01209078	II ^c^	Bacterial skin infection	[119]
hLF1-11	NCT00430469	I/II ^a^	Bacterial/fungal infections	[120,121]
Iseganan (IB-367)	NCT00118781; NCT00022373	III ^a^	Pneumonia/oral mucositis	[122]
LFF571	NCT01232595	II ^c^	*C. difficile*	[123]
LL-37	EUCTR2012-002100-41	II ^a^	Leg ulcers	[124]
LTX-109	NCT01803035; NCT01158235	I/II ^c^	MRSA/impetigo, antiviral	[125]
Mel4	ACTRN1261500072556	II/III ^c^	Contact lenses antimicrobial	[126]
Melittin	NCT02364349, NCT01526031	I/II ^c^	Inflammation	[127]
Murepavadin	EUCTR2017-003933-27-EE	II ^b^	*P. aeruginosa, K. pneumoniae*	[128]
Nal-P-113	ChiCTR-OIC-16010250	III ^c^	Periodontal disease	[129]
Neuprex^®^	NCT00462904	III ^a^	Pediatric meningococcemia	[107]
Nisin	NCT02928042; NCT02467972	n.a. ^c^	Gram-positive bacteria	[130]
Novexatin (NP213)	NCT02933879	II ^a^	Fungal nail infection	[131]
NVB-302	ISRCTN40071144	I ^a^	*C. difficile*	[107]
Omiganan	NCT00231153; NCT02456480	II/III ^c^	Antisepsis/catheter, Atopic dermatitis	[104]
OP-145	ISRCTN84220089	I/II ^c^	Chronic middle ear infection	[132]
PAC113	NCT00659971	II ^c^	Oral candidiasis	[133,134]
P60.4Ac	ISRCTN12149720	II ^c^	Chronic ear infections	[135]
Pexiganan (MSI-78)	NCT00563394; NCT00563433; NCT01590758; NCT01594762	III ^a^	Diabetic foot ulcers	[136]
PMX-30063	NCT01211470; NCT02052388	II ^c^	Acute bacterial skin infection	[137]
Polymyxin B	NCT00490477; NCT00534391	III ^d^	Gram-negative bacteria	[138]
Polymyxin E (Colistin)	NCT01292031; NCT02573064	III ^c^	*A. baumannii*/pneumonia	[139]
PXL01	NCT01022242	II/III ^c^	Postsurgical adhesions	[140,141]
SGX942(Dusquetide)	NCT03237325	III ^c^	Oral mucositis	[142,143]
Surotomycin (CB-315)	NCT01597505	III ^a^	*C. difficile*	[144]
XF-73(Exeporfinium chloride)	NCT03915470	II ^c^	*Staphylococcal* infection	[145]

^a^—Discontinued; ^b^—Ongoing; ^c^—Completed; ^d^—Unknown; n.a.—not applicable.

## Data Availability

Data is contained within the article.
